# Genome-Wide Identification and Characterization of the Medium-Chain Dehydrogenase/Reductase Superfamily of *Trichosporon asahii* and Its Involvement in the Regulation of Fluconazole Resistance

**DOI:** 10.3390/jof10020123

**Published:** 2024-02-01

**Authors:** Xiaoping Ma, Zhen Liu, Xiangwen Zeng, Zhiguo Li, Rongyan Luo, Ruiguo Liu, Chengdong Wang, Yu Gu

**Affiliations:** 1 Key Laboratory of Animal Disease and Human Health of Sichuan Province, College of Veterinary Medicine, Sichuan Agricultural University, Chengdu 611130, China; liuzhen@stu.sicau.edu.cn (Z.L.); 2022203060@stu.sicau.edu.cn (X.Z.); 2022303131@stu.sicau.edu.cn (Z.L.); lry9494@163.com (R.L.); liuruiguo@stu.sicau.edu.cn (R.L.); 2 China Conservation and Research Center for the Giant Panda, Chengdu 611800, China; wangchengdong@aliyun.com; 3 College of Life Sciences, Sichuan Agricultural University, Chengdu 611130, China

**Keywords:** *Trichosporon asahii*, the MDR superfamily, fluconazole, resistance

## Abstract

The medium-chain dehydrogenase/reductase (MDR) superfamily contains many members that are widely present in organisms and play important roles in growth, metabolism, and stress resistance but have not been studied in *Trichosporon asahii*. In this study, bioinformatics and RNA sequencing methods were used to analyze the MDR superfamily of *T. asahii* and its regulatory effect on fluconazole resistance. A phylogenetic tree was constructed using *Saccharomyces cerevisiae*, *Candida albicans*, *Cryptococcus neoformans,* and *T. asahii,* and 73 *MDRs* were identified, all of which contained NADPH-binding motifs. *T. asahii* contained 20 *MDRs* that were unevenly distributed across six chromosomes. *T. asahii MDRs* (*TaMDRs*) had similar 3D structures but varied greatly in their genetic evolution at different phylum levels. RNA-seq and gene expression analyses revealed that the fluconazole-resistant *T. asahii* strain upregulates xylitol dehydrogenase, and downregulated alcohol dehydrogenase and sorbitol dehydrogenase concluded that the fluconazole-resistant *T. asahii* strain was less selective toward carbon sources and had higher adaptability to the environment. Overall, our study contributes to our understanding of *TaMDRs*, providing a basis for further analysis of the genes associated with drug resistance in *T. asahii*.

## 1. Introduction

*Trichosporon asahii* (*T. asahii*) is a fungus belonging to the genus Trichosporon and is the most common and important conditionally pathogenic fungus among all Trichosporon species, causing disease in both humans and animals. It is the main causative agent of invasive, systemically disseminated trichosporonosis [1,2]. Trichosporonosis is a complex fungal infection that can involve multiple organs, is difficult to treat using antifungal drugs, is prone to recurrence, and is associated with a poor prognosis [3,4,5]. Triazole antifungals, such as fluconazole, have become the most commonly used antifungal drugs in clinics due to their high safety and efficacy [6]. However, in recent years, there have been increasing reports of azole resistance in clinical isolates of *T. asahii* and therapeutic failure after azole application. A number of clinical isolates have been found to be insensitive to fluconazole in in vitro drug sensitivity tests, resulting in a poor response to antifungal therapy in patients with invasive trichosporonosis [7,8].

The medium-chain dehydrogenase/reductase (MDR) superfamily is composed of alcohol dehydrogenases (ADH), cinnamyl alcohol dehydrogenases (CAD), sorbitol dehydrogenases (SDH), quinone oxidoreductases (QOR), and many other enzymes [9]. MDR proteins have two conserved structural domains: a GroES-like structural domain with the catalytic structural domain of the enzyme (ADH_N) and a typical Rossmann folded zinc-binding (ADH_zinc_N) coenzyme-binding domain [10,11]. ADHs are widely distributed in all types of organisms, are involved in organismal virulence, growth, metabolism, and resistance, and are the most frequently reported family in the MDR superfamily [12,13,14,15]. Recently, ADH has been found to be involved in fungal drug resistance. *ADH1* is differentially expressed in fluconazole-resistant and -sensitive strains of *Candida albicans* [16,17]. A clinical comparison of 20 clinical *C. albicans* strains isolated from patients with vulvovaginal candidiasis (VVC) and *C. albicans*-susceptible and -resistant strains revealed that *ADH1* expression was 10.63 to 17.61 times higher in the resistant strains than in the susceptible strains [18]. ADH1p is involved in fluconazole resistance in *C. albicans* and plays an important role in energy metabolism [19]. In addition to ADH proteins, SDH and xylitol dehydrogenases (XDH) are involved in energy metabolism. SDH play a fundamental role in polyol metabolism, and the sorbitol metabolic pathway is a key bypass for glycolysis during glucose metabolism [20]. Some microorganisms use sorbitol as an alternative carbon and energy source [21]. XDH is involved in xylose metabolism, and its metabolites enter the pentose phosphate pathway for translocation to produce energy [22].

Many MDR superfamily members have been identified and functionally analyzed in bacteria and plants; however, no such studies have been performed in fungi. We identified MDR superfamily members in *T. asahii* for the first time using bioinformatics and analyzed the gene structure, chromosomal location, conserved motifs, interspecies phylogenetic evolution, and three-dimensional (3D) structure of the encoded proteins, as well as the transcriptional expression of these *T. asahii MDRs* (*TaMDRs*). The aim of this study was to gain a comprehensive understanding of *TaMDRs* and preliminarily investigate their expression patterns in drug-resistant *T. asahii* strains.

## 2. Materials and Methods

### 2.1. Strain Material

Wild-type *T. asahii* strains (WT) and fluconazole-resistant strains (PB) were obtained from the Clinical Veterinary Medicine Laboratory of the College of Veterinary Medicine, Sichuan Agricultural University, China. WT and PB were used for RNA sequencing, with three replicates per group.

### 2.2. Acquisition of Genomic Information

The complete dataset of *T. asahii* was obtained from the China National Center for Bioinformation/Beijing Institute of Genomics, Chinese Academy of Sciences (GSA: CRA014197). *Cryptococcus neoformans*, *C. albicans,* and *Saccharomyces cerevisiae* genome and protein sequences were downloaded from the Ensembl fungi genome database (https://fungi.ensembl.org/index.html (accessed on 27 August 2023)). The cds, genome, gff, and pep files were selected for subsequent analysis.

### 2.3. Identification of MDR Superfamily Members

The hidden Markov models (HMM) of the zinc-binding dehydrogenase domain (ADH_Zinc_N, PF00107) and the alcohol dehydrogenase GroES-like domain (ADH_N, PF08240) were obtained from the Pfam database (http://pfam.xfam.org/ (accessed on 13 January 2023)). Sequences containing both the ADH_Zinc_N and ADH_N domains were screened and identified using HMMSEARCH (threshold e-value < 0.001). The Conserved Domain Database (CDD, https://www.ncbi.nlm.nih.gov/cdd (accessed on 1 September 2023)) was used to confirm the conserved domains of the collected sequences, and sequences containing ADH_Zinc_N and ADH_N domains were considered to belong to the MDR superfamily.

### 2.4. Analysis of the Protein Properties of TaMDRs

ExPASy (http://web.expasy.org/protparam/ (accessed on 13 September 2023)) was used to predict the basic properties of *MDR* gene-encoded proteins, including their length, isoelectric point (pI), and molecular weight (MW) [23]. Subcellular localization was predicted using WoLF PSORT (https://wolfpsort.hgc.jp/ (accessed on 13 September 2023)) [24].

### 2.5. Phylogenetic Analysis

To explore the evolutionary relationships of *TaMDRs*, *C. neoformans* (*CnMDR*), *C. albicans* (*CaMDR*), and *S. cerevisiae* (*ScMDR*) maximum likelihood phylogenetic evolutionary trees were constructed using Molecular Evolutionary Genetics Analyses (MEGA 7.0), and tree topology support was assessed by bootstrap analyses with 1000 replicates [25]. ClustalW 2.0.10 software was used for multiple sequence comparisons to assess the evolutionary relationships of *MDR* members among different species using default parameters. The WAG + G + I amino acid substitution model was used. Furthermore, the phylogenetic trees of *TaMDRs* were constructed using MEGA-X with maximum likelihood, the WAG + G amino acid model, and 1000 bootstrap replications. The constructed phylogenetic tree was annotated and visualized using EvolView-v2 (https://www.evolgenius.info/evolview/ (accessed on 27 September 2023)) [26].

### 2.6. Gene Structure and Conserved Motif Analysis

To analyze the *TaMDR*, *CnMDR*, *CaMDR,* and *ScMDR* gene structures and conserved motifs, the exon introns of MDR proteins were analyzed and visualized using the online Gene Structure Display Server tools (GSDS, http://gsds.cbi.pku.edu.cn/ (accessed on 2 October 2023)) [27]. The MEME program (http://alternate.meme-suite.org/tools/meme (accessed on 4 October 2023)) was used to identify conserved motifs in the *MDR* sequence, with the number of motifs set to 15 and the minimum and maximum widths set to 10 and 50, respectively [28].

### 2.7. Comparison of Multiple Sequences and Protein Structure Prediction of TaMDRs

*TaMDR* sequences were compared using ClustalW in MEGA software (version 7.0), and ESPript 3.0 (https://espript.ibcp.fr/ESPript/cgi-bin/ESPript.cgi (accessed on 18 October 2023)) was used to visualize the results [29]. Three-dimensional protein models were constructed using the SWISS-MODEL website (http://swis model.expasy.org (accessed on 12 November 2023)) [30]. The generated models were evaluated using SAVES v6.0 software (https://saves.mbi.ucla.edu/ (accessed on 15 November 2023)), which provides six methods evaluations simultaneously, three of which display a pass, indicating that the model is available. The protein structure was visualized using the SWISS-PDB viewer 4.10.

### 2.8. Collinearity Analysis

Genome-wide covariance analyses of *T. asahii*, *C. neoformans*, *C. albicans,* and *S. cerevisiae* were performed using the one-step MCScanX module of TBtools v2.012 software to obtain genome-wide replication events [31].

### 2.9. Analysis of the Localization of the TaMDR Gene on Chromosomes

To understand the chromosomal distribution of the *MDR* members in the *T. asahii* genome, the chromosomal location information of the *MDR* members was obtained from the Gene Structure Annotation Information file. Visualization was performed using TBtools software v 2.027 [31].

### 2.10. Analysis of TaMDR Expression Pattern in Fluconazole Resistance

Transcriptome sequencing was performed to investigate the expression patterns of *MDR* members in clinical isolates (WT) and fluconazole-resistant strains (PB) of *T. asahii*. The complete dataset has been submitted to the NCBI SRA database (accession number PRJNA941075). Gene expression levels were estimated as FPKM (fragments per kilobase of transcript per million mapped reads) values. Heatmaps of the transcript expression levels of differentially expressed *TaMDRs* were plotted using a heatmapper (http://www.heatmapper.ca/ (accessed on 30 November 2023)) [32].

### 2.11. Gene Expression Analysis

Total RNA from the WT and PB strains was extracted using the SteadyPure RNA Extraction Kit (Accurate Biotechnology (Hunan) Co., Ltd., Changsha, China) according to the manufacturer’s instructions. A cDNA template was synthesized from the RNA via reverse transcription using an Evo M-MLV RT Kit with clean gDNA (Accurate Biotechnology (Hunan) Co., Ltd., Changsha, China). Primer Premier 5 software was used to design gene-specific primers; 18s rRNA was used as the internal reference gene [33]. Each qPCR contained 10 uL SYBR Green ProTaq HS Premix (Accurate Biotechnology (Hunan) Co., Ltd., Changsha, China), 1 uL cDNA, and 0.4 µM of each gene-specific primer (Supplementary Table S1) at a final volume of 20 mL and was performed on a CFX96 real-time PCR system (BioRad, Hercules, CA, USA). The following reaction procedure was used: 95 °C for 30 s, followed by 40 cycles of 95 °C for 5 s, and 60 °C for 30 s. qRT-PCR data were analyzed using the 2^−∆∆Ct^ method to calculate the relative expression levels. All assays were performed in triplicate [34]. All statistical analyses were performed using GraphPad Prism 8.01 (GraphPad Software Inc., La Jolla, CA, USA).

## 3. Results

### 3.1. Identification and Basic Properties of TaMDRs

A HMM was used to identify *MDR* superfamily members in *T. asahii*, and 20 genes containing both the ADH_Zinc_N and ADH_N domains were identified and named *TaMDR_1*–*TaMDR_20* (Table 1). The predicted lengths of the proteins encoded by the *TaMDRs* ranged from 308 to 416 amino acids (aa). The molecular weights ranged from 32,819.7 Da (*TaMDR_6*) to 44,464.9 Da (*TaMDR_1*). The isoelectric point values of these *TaMDRs* ranged from 5.74 to 8.08 and included 5 basic and 15 acidic proteins. Subcellular localization predictions suggested that 16 TaMDR proteins were localized in the cytoplasm, and 4 proteins were localized in the mitochondria.

### 3.2. Phylogenetic Analysis

**Figure 1:**
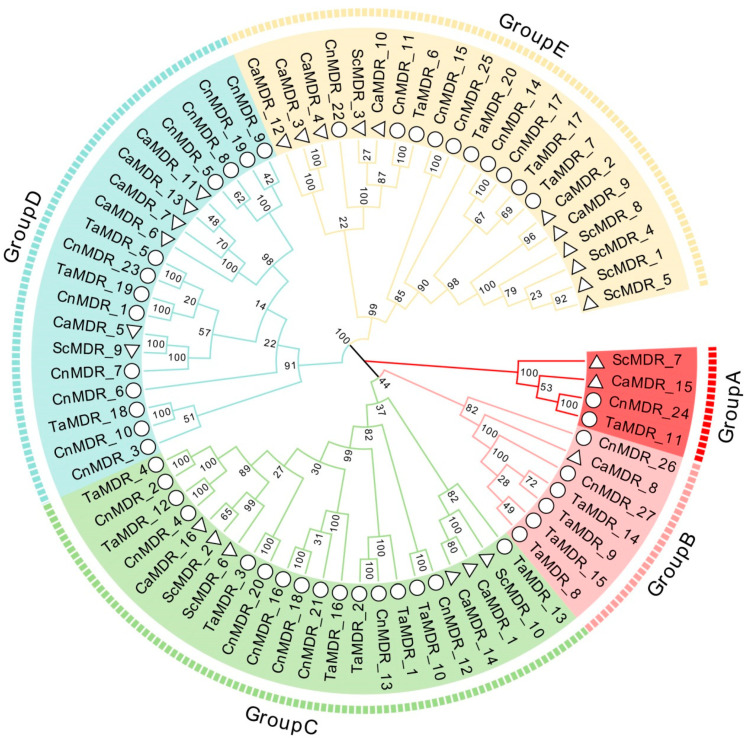
Phylogenetic analysis of the *Trichosporon asahii*, *Cryptococcus neoformans*, *Candida albicans,* and *Saccharomyces cerevisiae* medium-chain dehydrogenase/reductase (*MDR*) superfamily members. Arcs of different colors indicate different groups. Circles and triangles represent *MDRs* of the Basidiomycota (*T. asahii* and *C. neoformans*) and Saccharomycotina (*C. albicans* and *S. cerevisiae*), respectively.

To understand the evolutionary relationships between the proteins encoded by *TaMDRs*, we selected three model fungal species, *S. cerevisiae*, *C. albicans,* and *C. neoformans*, to construct a phylogenetic tree with the *TaMDRs* (Figure 1, Supplementary Table S2). A total of 73 *MDRs* were identified in the four fungal strains, including 20 *TaMDRs* from *T. asahii*, 27 *CnMDRs* from *C. neoformans*, 16 *CaMDRs* from *C. albicans*, and 10 *ScMDRs* from *S. cerevisiae*. Phylogenetic analysis was used to categorize the predicted *MDRs* into five major branches (Group A–E), revealing high homology between *C. neoformans* and *T. asahii* and between *C. albicans* and *S. cerevisiae*.

**Figure 2:**
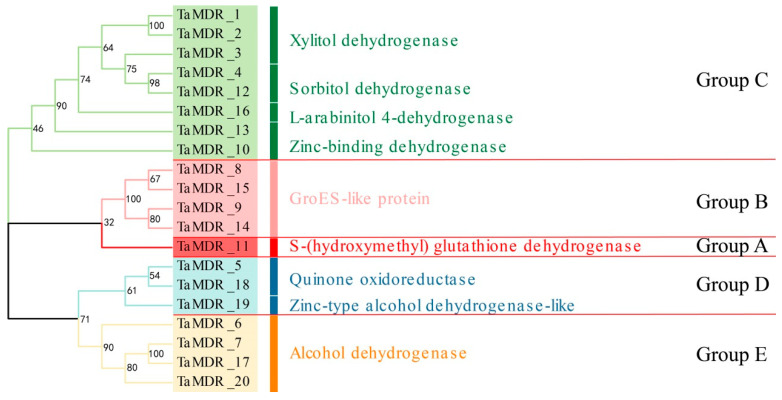
Phylogenetic analysis of the *T. asahii MDRs* (*TaMDRs*). Arcs of different colors indicate different groups.

In addition, a phylogenetic tree of *TaMDRs* was constructed, which was divided into five branches, as shown in Figure 1, and the relevant annotation information for each gene was marked (Figure 2). Among the 20 *TaMDRs* identified, only *TaMDR_11* encoding S-(hydroxymethyl) glutathione dehydrogenase was present in Group A. Group B encoded a GroES-like protein, which is a marker protein encoded by the ADH_N domain. Group C contained three xylitol dehydrogenases, two sorbitol dehydrogenases, and one L-arabinitol 4-dehydrogenase. Group D included two quinone oxidoreductases, two zinc-bound dehydrogenases, and one zinc-type alcohol dehydrogenase-like protein. A total of four alcohol dehydrogenases were identified.

### 3.3. Multiple Sequence Comparisons of TaMDRs

**Figure 3:**
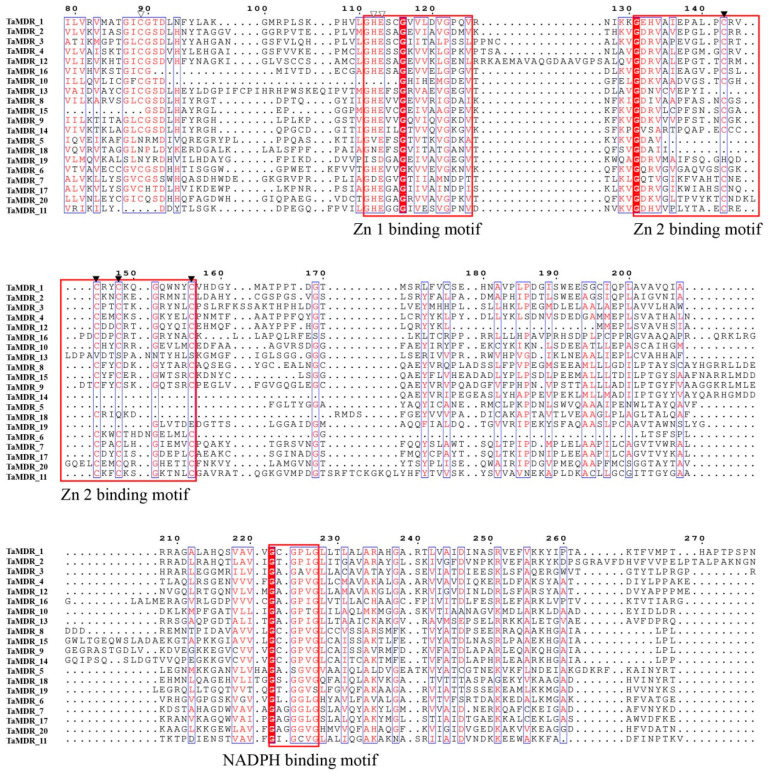
Protein sequence alignment of all identified *TaMDRs*. The red background means the amino acids are identical. The red frames represent the binding motifs of Zn1, Zn2, and NADPH, respectively. White triangles represent catalytic zinc ligand residues, and black triangles represent structural zinc ligand residues.

Multiple sequence comparisons were performed for the 20 proteins of the *TaMDRs* (Figure 3). Zn 1-binding motifs, Zn 2-binding motifs, and NADPH-binding motifs are highly important binding motifs for *MDRs*. The Zn1-binding motif [GHE(x)2G(X)5G(X)2V] contains a catalytic zinc amino acid coordination residue, and the Zn-binding motif [GD(X)9,10C(X)2C(X)2C(X)7C] contains a structural zinc amino acid coordination residue. Groups A and E have a conserved Zn 1-binding motif and Zn 2-binding motif. Group D lacks both Zn-1-binding motifs and Zn-2-binding motifs. The NADPH-binding domain [GXG(X)2G] was highly conserved in all *TaMDRs*.

### 3.4. Gene Structure and Protein Motif Analysis of the MDRs

**Figure 4:**
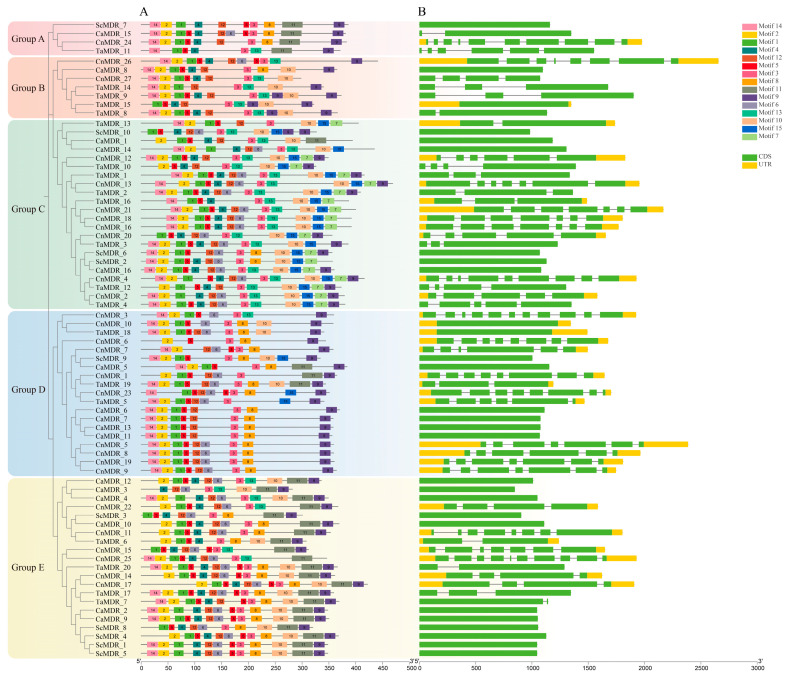
Phylogenetic tree, gene structure, and conserved motifs of *MDRs*. (**A**) Phylogenetic tree and motif analysis; the length of the solid line represents the length of the protein sequence. Colored boxes represent different motifs. (**B**) Gene structure analysis of the *MDRs*. Yellow boxes indicate untranslated regions (UTR) regions; green boxes indicate CDS regions; introns are indicated by black lines. The scale at the bottom is in base pairs (bp).

To study the structural diversity of *MDRs*, their exon–intron structures and motif distributions were analyzed (Figure 4B). The coding sequences (CDSs) of Groups A-E ranged from 1 to 11. A total of 26 genes were identified in *C. albicans* and *S. cerevisiae*; only *CaMDR_15* had two CDSs, whereas the remaining 25 genes had one CDS. Approximately 26 genes had no untranslated regions (UTRs) at the C-terminus or N-terminus. A total of 47 genes were identified in *T. asahii* and *C. neoformans*, of which three genes had only one CDS region (*TaMDR_15*, *TaMDR_18,* and *CnMDR_10*). Genes containing three or seven CDS regions were the most abundant, followed by genes containing four CDS regions.

**Figure 5:**
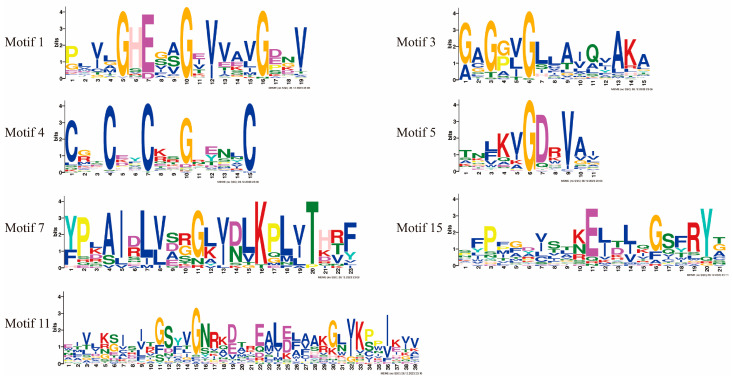
Basic composition of *MDR motifs*.

Next, we analyzed the conserved motifs present in the *MDRs* and found a total of 15 conserved motifs in genes with higher homology and similar protein motif compositions (Figure 4A and Figure 5, and Supplementary Figure S1). Motifs 1, 2, 3, 5, and 12 were detected in most of the *MDR* gene sequences. Motif 1 is a Zn 1-binding motif that was detected in 63 *MDRs*. Motif 3 (an NADPH-binding motif) was detected in all *MDRs*. Together, motifs 4 and 5 form the Zn 2-binding motif, with motif 4 containing four cysteine coordination residues of the zinc structure. Motif 5 was not detected in *Sc_MDR7*, and 51 genes contained motif 4. Motif 11 was the characteristic motif of Groups A and E. Motifs 7 and 15 were the characteristic motifs of Group C. Motifs 4 and 7 were absent in Group D, similar to the results of *TaMDRs* multiple sequence comparisons.

### 3.5. D Structure Prediction of TaMDRs

**Figure 6:**
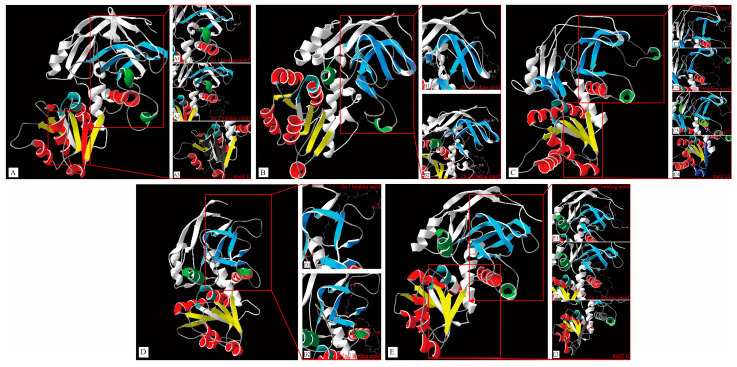
The protein 3D structures in *TaMDRs*. (**A**) *TaMDR_11*; (**B**) *TaMDR_9*; (**C**) *TaMDR_12*; (**D**) *TaMDR_19*; (**E**) *TaMDR_17*. The red α-helix and the yellow β-sheet form the ADH_Zinc_N domain. The green α-helix and the blue β-sheet form the ADH_N domain. The blue-green region represents the NADPH-binding motifs. **A1**–**E1** and **A2**–**E2** represent the Zn1- and Zn2-binding motifs, respectively. **A3** and **E3** represent motif 11, **C3** represents motif 7, and **C4** represents motif 15.

*TaMDR_11*, *TaMDR_9*, *TaMDR_12*, *TaMDR_19,* and *TaMDR_17* genes were selected as representatives to analyze the related 3D structures (Figure 6). The Zn 1-binding motifs of *TaMDR_11*, *TaMDR_9* and *TaMDR_12* belonged mainly to the β-sheet (Figure 6(A1,B1,C1)), and those of *TaMDR_19* and *TaMDR_17* belonged to the β-sheet and the random coil (Figure 6(D1,E1)). The Zn 2-binding motifs in all five genes were located in a β-sheet, an α-helix, and an irregular coil (Figure 6(A2,B2,C2,D2,E2)). The NADPH-binding motif was located in the middle of the ADH_N and ADH_Zinc_N domains and was immediately adjacent to the ADH_Zinc_N domain (Figure 6A–E). Motif 11 is a characteristic motif in both Groups A and E, but in the 3D structure, it exhibited a large disparity; motif 11 in *TaMDR_11* consisted of two β-sheets and one α-helix, while in *TaMDR_17,* it consisted of one β-sheet and two α-helixes (Figure 6(A3,E3)). Motif 15 and motif 7 were the characteristic sequences in Group C, and both motifs consisted of one β-sheet and one α-helix (Figure 6(C3,C4)).

### 3.6. Chromosomal Localization of TaMDRs

**Figure 7:**
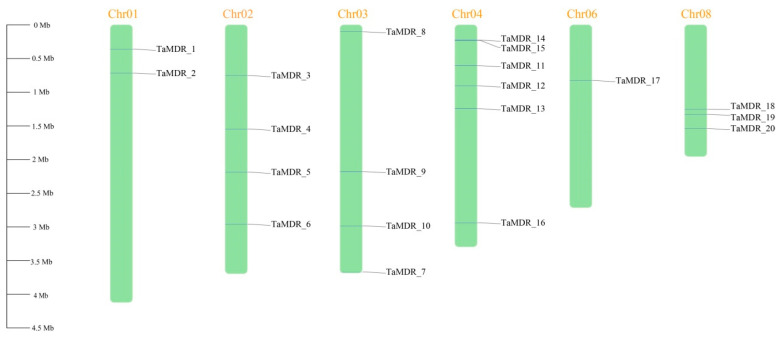
Distribution of *TaMDR* genes on the chromosomes of *T. asahii*. The chromosomes are represented by green barred boxes, and different horizontal lines on the chromosomes represent genes at different locations. The scale on the left is in megabases (Mb).

According to the whole-genome sequencing results, *T. asahii* has eight chromosomes, and *TaMDRs* are unevenly distributed among six chromosomes (Figure 7). Six genes were distributed on chromosome 4, followed by four on chromosomes 2 and 3. The least number of genes was distributed on chromosome 6, with only one gene.

### 3.7. Collinearity Analysis

**Figure 8:**
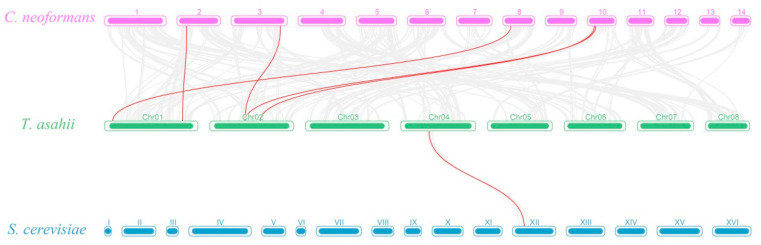
Comparative linear relationship of *MDRs* in *C. neoformans*, *T. asahii,* and *S. cerevisiae.* 1–14 represents the chromosome number of *C. neoformans,* and I–XVI represents the chromosome number of *S. cerevisiae*.

We did not observe intraspecific collinearity in *T. asahii*, and further comparative collinearity maps were constructed at the genomic level for four fungi: Basidiomycota (*T. asahii* and *C. neoformans*) and Saccharomycotina (*S. cerevisiae* and *C. albicans*) (Figure 8 and Supplementary Figure S2). Two *TaMDRs* on chromosome 1 (*TaMDR_1*) and chromosome 2 (*TaMDR_4*) were co-linked with the MDRs of *C. neoformans* (*CnMDR_7*, *CnMDR_12*, *CnMDR_18*, *CnMDR_19*, and *CnMDR_20*). Only one *TaMDR* on chromosome 4 (*TaMDR_13*) exhibited a collinear relationship with *S. cerevisiae* (*ScMDR_6*), whereas there were no collinear genes with *C. albicans*. All collinear genes were present in Groups C and D (Figure 1). In addition, with the exception of three *MDRs*, *S. cerevisiae* and *C. albicans* had very few genes that were collinear with *T. asahii*, whereas *C. neoformans* had many collinearities with *T. asahii*.

### 3.8. Transcription and Expression of TaMDRs in Drug-Resistant Strains

**Figure 9:**
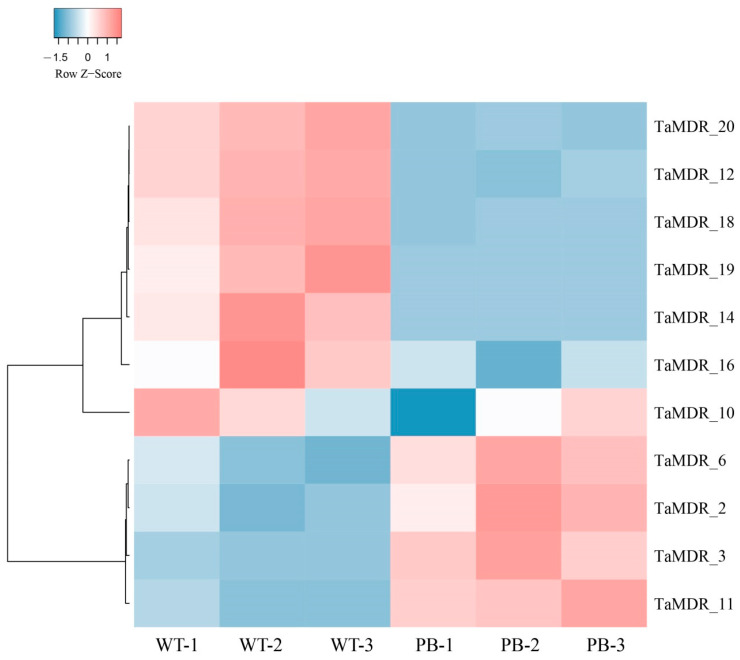
Heatmap of *MDR* transcript expression levels in wild-type (WT) and resistant (PB) *T. asahii* strains, expressed as Log2FC values. Colored blocks indicate upregulated (red) or downregulated (blue) levels. White blocks indicate genes that were not differentially expressed.

Ribonucleic acid sequencing (RNA-seq) was performed on WT and PB strains, and the |Log2 fold change (Log2FC)| > 0.5 and false discovery rate < 0.05 were used to identify differentially expressed genes (DEGs). Eleven DEGs were differentially altered in the resistant strain, and seven were downregulated in the resistant strain (Figure 9). *TaMDR_19* and *TaMDR_20* exhibited the most significant differences in expression among the resistant strains and were downregulated by 5.8- and 5-fold, respectively. *TaMDR_3* was the most significantly upregulated gene, with a 2.25-fold upregulation.

### 3.9. QRT-PCR Analysis of TaMDRs in Fluconazole-Resistant Strains

**Figure 10:**
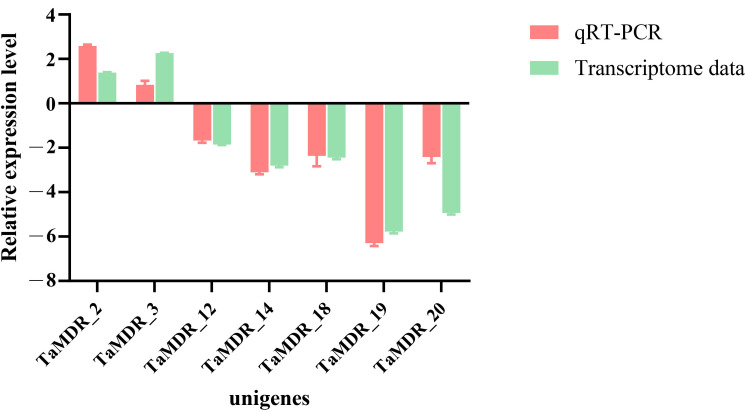
Gene expression of *TaMDRs* in WT and PB strains. The x-axis indicates the codes of the seven differentially expressed genes (DEGs) in *T. asahii,* and the y-axis indicates the gene expression levels. The values are expressed as mean ± SDs. Green bars represent transcriptome results, and red bars represent qRT-PCR results.

To further investigate the differences in *TaMDR* expression in fluconazole-resistant *T. asahii* strains, qRT-PCR was performed to verify the expression of seven genes with significantly different expression levels (Figure 10). The relative expression levels of *TaMDR_2* and *TaMDR_3* were upregulated in the resistant strain compared to those in the wild-type strain, with *TaMDR_3* exhibiting the highest expression level (2.58-fold higher than that in the wild-type strain). In contrast, *TaMDR_12*, *TaMDR_14*, *TaMDR_18*, *TaMDR_19*, and *TaMDR_20* were significantly downregulated in the resistant strains, especially *TaMDR_19*, which exhibited a 6.3-fold decrease in expression compared to that in the wild-type strain. Overall, the qRT-PCR results were consistent with the transcriptomic data.

## 4. Discussion

*Trichosporon asahii* is the causative agent of invasive trichosporonosis, and fluconazole-resistant *T. asahii* strains make its clinical treatment a major challenge [35]. In this study, we explored, for the first time, the composition of the *MDR* superfamily members of *T. asahii* and their expression patterns in drug-resistant strains, providing new insights into the role of *MDRs* in the drug resistance of *T. asahii*.

*S. cerevisiae*, *C. albicans*, and *C. neoformans* are model fungal species, of which *C. neoformans* and *C. albicans* are common pathogenic fungi in humans and animals [36,37,38]. *C. neoformans* and *T. asahii* belong to Basidiomycota, whereas *C. albicans* and *S. cerevisiae* belong to Saccharomycota; therefore, these three fungi were selected to jointly explore the genetic evolutionary relationships of *TaMDRs*. In this study, Basidiomycota (*T. asahii* and *C. neoformans*) were shown to have 20 and 27 *MDRs*, respectively, whereas Saccharomycotina (*C. albicans* and *S. cerevisiae*) had 16 and 10 *MDRs*, respectively. Basidiomycota can thus be inferred to possess a higher number of *MDRs* than Saccharomycotina. The motif distribution and gene structure of fungal *MDRs* associated with phylogenetic trees are important tools for sequence characterization in genetic studies. Fungi in the same phylum exhibited high homology, and genes within the same group had similar protein motifs. *MDRs* in Basidiomycota had a more complex gene structure than those in Saccharomycotina, with most *MDRs* in *C. albicans* and *S. cerevisiae* having only a single CDS region, whereas *T. asahii* and *C. neoformans* were more inclined to have multiple CDS regions. A simpler gene structure for Saccharomycotina has also been reported in studies of the Cyclophilin family [39]. In addition, *T. asahii* exhibited a greater collinearity with *C. neoformans* and very little collinearity with *C. albicans* and *S. cerevisiae*. Our results suggest that fungi have evolved with significant evolutionary differences at the phylum level.

We constructed a phylogenetic tree for *TaMDRs*, and the S-(hydroxymethyl) glutathione dehydrogenase (FADH) in Group A was considered as the origin of the alcohol dehydrogenase family, which, together with alcohol dehydrogenase, constitutes the ADH family [40]. Motif 11 is a characteristic motif of Groups A and E and is also present in the PRK09422 family within the MDR superfamily (alcohol-active dehydrogenase/acetaldehyde-active reductase). It is detected in Group B’s *CaMDR_1* and Group C (*TaMDR_19*, *CnMDR_1*, and *CaMDR_5*), but only *TaMDR_19* and *CnMDR_1* are annotated as alcohol dehydrogenase. Therefore, MDRs containing motif 11 are inferred to possess aldehyde reductase activity. Group C was composed of xylitol dehydrogenase, sorbitol dehydrogenase, L-arabinitol 4-dehydrogenase, and zinc-binding dehydrogenase, which belong to the PDH (polyol dehydrogenase) family of the MDR superfamily [11]. Characteristic motifs 7 and 15 in Group C have both been identified in zinc-dependent alcohol dehydrogenase-like family proteins. Therefore, Group C MDRs may possess the ability to catalyze NAD(P)(H)-dependent reversible conversion of alcohols to their corresponding aldehydes. In Group D, *TaMDR_5*, *TaMDR_18,* and *TaMDR_19* contained QOR-specific sites. However, *TaMDR_19* was annotated as a zinc-type alcohol dehydrogenase-like protein instead of a quinone oxidoreductase, leading to the inference that *TaMDR_19* is an atypical alcohol dehydrogenase. In the multiple sequence comparisons, these genes did not have complete catalytic or structural zinc-binding sites, and we speculated that *TaMDR_19* clustered in Group D, which is consistent with the characteristic of Group D lacking the Zn 1- (motif 1) and Zn 2-binding motifs (motifs 4 and 5). Here, we believed that although the Basidiomycota (*T. asahii* and *C. neoformans*) and the Saccharomycotina (*C. albicans* and *S. cerevisiae*) exhibit genetic structural differences, the types and distributions of motifs are similar within the same group.

MDR proteins consist of two domains (ADH_N and ADH_Zinc_N), where the C-terminal domain is a typical Rossmann fold, which consists of six parallel β-sheets with two α-helices on each side [41]. The N-terminal domain consists of antiparallel β-sheets and surface-positioned α-helices, with long-range homology to the GroES structure [42]. This is consistent with our predicted 3D structure of some *MDRs*. Zinc-dependent alcohol dehydrogenases usually form dimers (higher plants and mammals) or tetramers (yeast and bacteria), each of which has two tightly bound zinc atoms per subunit, a catalytic zinc at the active site, and a structural zinc in the lobe of the catalytic domain in the Zn 1 (motif 1)- and Zn 2 (motif 4 and motif 5)-binding motifs, respectively [43]. In *S. cerevisiae*, the catalytic zinc is coordinated in two ways: a “classical” coordination of Cys-43, His-66, and Cys-153 and an “alternative” coordination of Cys-43, His-66, Glu-67, and Cys-153. Catalytic zinc coordination is relatively flexible, leading to the replacement of zinc-bound water with alcohols or aldehydes, thus contributing to the catalytic process of alcohols and aldehydes [44,45]. With the exception of Groups C and D, the catalytic zinc shown in the multiple sequence comparisons of *TaMDRs* is the binding mode of “classical” coordination. In plants, the amino acid coordination residues of structural zinc are four cysteine residues located in the Zn 2-binding motif [46]. *TaMDR* proteins had similar structural zinc coordination residues. The side chain of the residues of the structural zinc interacts with the residues of the coenzyme-binding structural domain of another monomer, thus linking multiple monomers into a dimeric or tetrameric structure, while the NADPH motif site is a characteristic sequence for coenzyme binding, and the typical Rossmann fold acts as a coenzyme-binding domain, where the coenzyme binds at the carboxy-terminal end [44].

MDRs are also involved in the regulation of fungal carbon sources. Both aerobically and anaerobically, *S. cerevisiae* Adh1p can use glucose as a carbon source to produce ethanol and NAD, whereas *ADH2* in the cytoplasm can utilize ethanol as a carbon source to catalyze the conversion of ethanol to acetaldehyde under anoxic conditions [47]. *Candida maltosa* is able to ferment xylose, with *ADH1* and *ADH2* promoting xylose metabolism [48]. Under oxygen-limited conditions, *ADH1* is absent, and the growth of *Pichia stipitis* in the xylose medium is inhibited [49]. Cytoplasmic *TaMDR_2* and mitochondrial *TaMDR_3* were identified as xylitol dehydrogenases and were significantly upregulated in drug-resistant *T. asahii* strains. XDH is involved in xylose metabolism; xylulose produced by oxidation is phosphorylated and transported via the pentose phosphate pathway [50]. The pentose phosphate pathway produces NADPH in the aerobic phase, enters the glycolytic pathway in the anaerobic phase, and provides energy to the body in anaerobic environments. The significant upregulation of *TaMDR_2* and *TaMDR_3* in *T. asahii* provided more energy and enhanced the resistance of the resistant strain to anaerobic environments.

MDRs are involved in a variety of mechanisms, including growth and energy metabolism, under aerobic and anaerobic conditions [51]. Alcohol dehydrogenase catalyzes the interconversion of alcohols and aldehydes, which play an important role in the exposure of organisms to adverse stresses [15,52]. Adh3p is located in the mitochondria and participates in redox shuttling in *S. cerevisiae,* where it is formed during biosynthetic reactions and transported to the cytoplasm [53]. *TaMDR_19* and *TaMDR_20* were identified as ethanol dehydrogenases that were significantly downregulated in *T. asahii*. In addition, sorbitol dehydrogenase is involved in the reversible NAD conversion of D-sorbitol to D-fructose and forms a part of the sorbitol pathway, which is a key bypass for glycolysis during glucose metabolism [20]. *TaMDR_12* was identified as a sorbitol dehydrogenase that was significantly downregulated in the resistant strain. In fungal drug resistance mechanisms, the expulsion of drugs through efflux pumps, alterations in cell membrane permeability, and maintenance of cell wall integrity necessitate energy consumption [54,55]. In drug-resistant strains of *C. albicans*, the expression of *ADH1* is positively correlated with the expression of efflux pump genes *CDR1* and *CDR2* [18]. However, energy metabolism decreases after acquiring the resistant strain compared to the wild-type strain. Therefore, the wild-type strain is inferred to require more energy for the maintenance of normal metabolism than the resistant strain, and the energy requirement is relatively low; therefore, the resistant strain is more environmentally adaptive. Due to the relatively few studies on *T. asahii* and the limited transcriptome data, the broader exploration of *TaMDRs* is limited. Therefore, more transcriptome data related to carbon source changes or energy metabolism will contribute to a better understanding of the regulatory role of the MDR superfamily of the fluconazole-resistance *T. asahii*. This will hopefully be realized in future experiments.

## 5. Conclusions

Twenty *MDRs* were identified in the *T. asahii* genome, which were unevenly distributed on six chromosomes. The genetic evolution of *TaMDRs* varied greatly at the phylum level. *TaMDRs* had similar 3D structures, and all had NADPH-binding motifs. Fluconazole-resistant *T. asahii* strains upregulated xylitol dehydrogenase and participated in xylitol energy metabolism. However, the expression of both alcohol dehydrogenase and sorbitol dehydrogenase was downregulated compared to that in the wild-type strain, indicating that fluconazole-resistant *T. asahii* strains had lower carbon source selectivity, lower energy requirements for self-maintenance, and higher adaptability to the environment. In conclusion, our study fills a gap in the MDR superfamily in *T. asahii* and provides a basis for further analysis of genes associated with drug resistance in *T. asahii*.

## Figures and Tables

**Table 1:** Detailed information of the MDR superfamily members identified in *T. asahii*.

Gene	Gene Accession No	Length	MW (Da)	PI	Subcellular Localization
TaMDR_1	evm.model.Chr01.121	416	44,464.9	6.78	cytoplasm
TaMDR_2	evm.model.Chr01.194	409	43,551.6	6.64	cytoplasm
TaMDR_3	evm.model.Chr02.245	386	40,935.1	6.96	cytoplasm
TaMDR_4	evm.model.Chr02.483	380	40,665.7	5.65	cytoplasm
TaMDR_5	evm.model.Chr02.694	341	37,556.9	7.90	cytoplasm
TaMDR_6	evm.model.Chr02.955	308	32,819.7	7.39	cytoplasm
TaMDR_7	evm.model.Chr03.1119	369	39,170.8	7.19	cytoplasm
TaMDR_8	evm.model.Chr03.31	366	39,926.4	5.89	mitochondrial
TaMDR_9	evm.model.Chr03.653	373	40,057.9	6.24	cytoplasm
TaMDR_10	evm.model.Chr03.899	326	35,447.7	5.98	cytoplasm
TaMDR_11	evm.model.Chr04.188	371	39,685.2	6.50	cytoplasm
TaMDR_12	evm.model.Chr04.287	373	39,975.8	6.83	mitochondrial
TaMDR_13	evm.model.Chr04.387	405	43,332.5	7.84	mitochondrial
TaMDR_14	evm.model.Chr04.73	340	36,507.9	6.13	cytoplasm
TaMDR_15	evm.model.Chr04.76	322	34,674.5	5.69	mitochondrial
TaMDR_16	evm.model.Chr04.938	387	41,339.5	8.08	cytoplasm
TaMDR_17	evm.model.Chr06.247	359	38,170.5	5.74	cytoplasm
TaMDR_18	evm.model.Chr08.307	341	36,122.1	6.25	cytoplasm
TaMDR_19	evm.model.Chr08.320	344	36,829.0	6.96	cytoplasm
TaMDR_20	evm.model.Chr08.391	366	38,786.3	6.18	cytoplasm

## Data Availability

The raw sequence data of *T. asahii* reported in this paper have been deposited in the Genome Sequence Archive at the National Genomics Data Center, China National Center for Bioinformation/Beijing Institute of Genomics, Chinese Academy of Sciences (GSA: CRA014197), and are publicly accessible at https://ngdc.cncb.ac.cn/gsa (accessed on 29 December 2023).

## Supplementary Materials

The following supporting information can be downloaded at https://www.mdpi.com/article/10.3390/jof10020123/s1, Figure S1: Basic composition of motifs for MDRs; Figure S2: Comparative linear relationship of MDRs in *C. albicans*, *T. asahii*, and *S. cerevisiae*; Table S1: Fluorescent quantitative primers for *TaMDRs*; Table S2: Detailed information of alcohol dehydrogenase proteins (MDRs) identified in *C. neoformans*, *C. albicans*, and *S. cerevisiae*.
