# Supplementary material for: Genome-Wide Identification and Characterization of the Medium-Chain Dehydrogenase/Reductase Superfamily of Trichosporon asahii and Its Involvement in the Regulation of Fluconazole Resistance
Source: J Fungi (Basel). 2024 Feb 1;10(2):123. doi: 10.3390/jof10020123 (PMC10889790; doi:10.3390/jof10020123)
Supplement: Supplementary file 1 [file jof-10-00123-s001.zip › jof-2834103-supplementary.pdf]

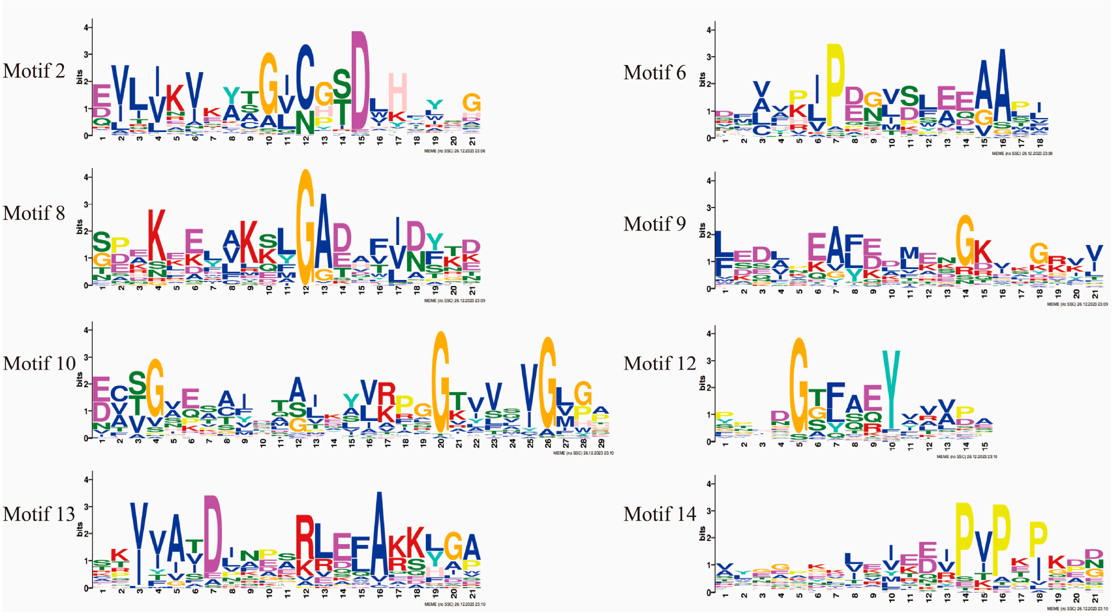

Supplementary Figure S1. Basic composition of motifs for *MDRs*.

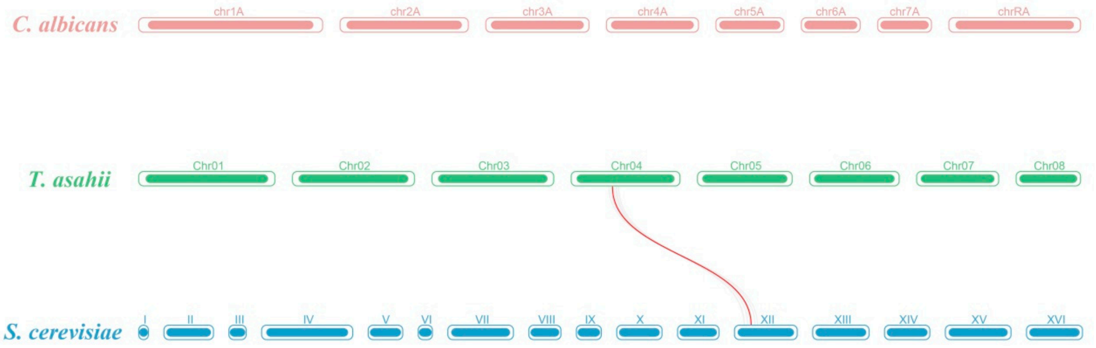

Supplementary Figure S2. Comparative linear relationship of *MDRs* in *C. albicans, T. asahii* and *S. cerevisiae.*

Supplementary Table S1. Fluorescent quantitative primers for *TaMDRs*

Primers	Sequence (5’-3’)
18s rRNA-F	TCTTTCTTGATTTTGTGGGTGG
18s rRNA-R	TCGATAGTCCCTCTAAGAAGTG
TaMDR_2-F	GCAAAGAGGGACGCATGAAC
TaMDR_2-R	AGGGCGAAGTAACGAGACAG
TaMDR_3-F	CGTGGTGAAGGGAAGGGGAC
TaMDR_3-R	CGCGAGCTGCATACAGGTCT
TaMDR_12-F	CACTGGCATCTGTGGTTCCG
TaMDR_12-R	CTTCCTCCTCAGGTTTTCGC
TaMDR_14-F	CGACCTCCCCAAGACCATGA
TaMDR_14-R	GCGACGCTTCTCTATTCCAA
TaMDR_18-F	ACCGAGAAGACGATGAAGGC
TaMDR_18-R	AATGGCGGGGAAAGGGAAGG
TaMDR_19-F	TGGCCAGACTGTCGTTACCC
TaMDR_19-R	TTCTTCAGCATCGCCTCCTT
TaMDR_20-F	CGGCACCTACACCTCATACC
TaMDR_20-R	GAGCCACTCGCCCTTCTTCA

Supplementary Table S2. Detailed information of alcohol dehydrogenase proteins (MDRs) identified in *C. neoformans, C. albicans* and *S. cerevisiae*.

Gene accession No	gene	Gene accession No	Rename
AAW40643	CnMDR_1	C1_04750W_A-T-p1	CaMDR_1
AAW40720	CnMDR_2	C1_08330C_A-T-p1	CaMDR_2
AAW40747	CnMDR_3	C2_04470W_A-T-p1	CaMDR_3
AAW40827	CnMDR_4	C2_04480W_A-T-p1	CaMDR_4
AAW41228	CnMDR_5	C2_07070W_A-T-p1	CaMDR_5
AAW41384	CnMDR_6	C2_10180W_A-T-p1	CaMDR_6
AAW41486	CnMDR_7	C4_06280C_A-T-p1	CaMDR_7
AAW41690	CnMDR_8	C5_02690W_A-T-p1	CaMDR_8
AAW41745	CnMDR_9	C5_05050W_A-T-p1	CaMDR_9
AAW42436	CnMDR_10	C6_02480W_A-T-p1	CaMDR_10
AAW42554	CnMDR_11	C6_04410C_A-T-p1	CaMDR_11
AAW42578	CnMDR_12	CR_02070C_A-T-p1	CaMDR_12
AAW42610	CnMDR_13	CR_03280W_A-T-p1	CaMDR_13
AAW43486	CnMDR_14	CR_05340C_A-T-p1	CaMDR_14
AAW44042	CnMDR_15	CR_10250C_A-T-p1	CaMDR_15
AAW44418	CnMDR_16	CR_10840C_A-T-p1	CaMDR_16
AAW44464	CnMDR_17	EGA84852	ScMDR_1
AAW45159	CnMDR_18	EGA85039	ScMDR_2
AAW45953	CnMDR_19	EGA85208	ScMDR_3
AAW45971	CnMDR_20	EGA85365	ScMDR_4
AAW46366	CnMDR_21	EGA85524	ScMDR_5
AAW46372	CnMDR_22	EGA85795	ScMDR_6
AAW46551	CnMDR_23	EGA87706	ScMDR_7
AAW46801	CnMDR_24	EGA87897	ScMDR_8
AAW46840	CnMDR_25	EGA88117	ScMDR_9
AAW46966	CnMDR_26	EGA88159	ScMDR_10
AAW47118	CnMDR_27		
